# Morphological and Postural Changes in the Foot during Pregnancy and Puerperium: A Longitudinal Study

**DOI:** 10.3390/ijerph18052423

**Published:** 2021-03-02

**Authors:** Monserrat Alcahuz-Griñan, Pilar Nieto-Gil, Pedro Perez-Soriano, Gabriel Gijon-Nogueron

**Affiliations:** 1 Department of Nursing and Podiatry, University of Valencia, 46010 Valencia, Spain; montse.alcahuz@uv.es (M.A.-G.); pilar.nieto@uv.es (P.N.-G.); 2 Research Group in Sport Biomechanics, Department of Physical Education and Sports, University of Valencia, 46010 Valencia, Spain; pedro.perez-soriano@uv.es; 3 Department of Nursing and Podiatry, Faculty of Health Sciences, University of Malaga, 29071 Malaga, Spain; 4 Biomedical Research Institute (IBIMA), 29010 Malaga, Spain

**Keywords:** pregnancy puerperium, foot, pedograph, foot posture index

## Abstract

The aim of this study is to observe the morphological and postural changes to the foot that take place during pregnancy and the puerperium. **Method:** In this descriptive, observational, longitudinal study, we analysed 23 pregnant women, with particular attention to morphological and postural aspects of the foot, at three time points during and after pregnancy: in weeks 9–13 of gestation, weeks 32–35 of gestation and weeks 4–6 after delivery. The parameters considered were changes in foot length, the Foot Posture Index (FPI) and the Hernández Corvo Index, which were analysed using a pedigraph and taking into account the Body Mass Index (BMI). The same procedure was conducted in each review. **Results:** The statistical analyses obtained for each foot did not differ significantly between the three measurement times. A pronator-type footprint was most frequently observed during the third trimester of pregnancy; it was predominantly neutral during the postpartum period. Statistically significant differences between the measurement times were obtained in the right foot for cavus vs. neutral foot type (between the first and third trimesters and also between the first trimester and the puerperium) (in both cases, *p* < 0.0001). **Conclusions:** Foot length increases in the third trimester and returns to normal in the puerperium. According to FPI findings, the third trimester of pregnancy is characterised by pronation, while the posture returns to neutrality during the postpartum period. During pregnancy, the plantar arch flattens, and this persists during the puerperium. The incidence of cavus foot increases significantly in the third trimester and in the puerperium.

## 1. Introduction

During the puerperium, i.e., the 6–8 weeks following childbirth, a series of physiological changes and weight loss occur until the maternal organism recovers its pregestational state [1,2]. Musculoskeletal modifications are usually resolved by the end of the sixth week of the puerperium, with the cessation of hormonal activity [3]. The relaxin hormone can produce the changes of the feet regarding length and width [4], and the increase of size shoes can produce the edemas in the feet [5].

During pregnancy, posture and gait are altered; the centre of gravity moves forward, increasing lumbar lordosis, while the head and trunk are borne further back. This change impacts on gait, as the body sways laterally and the legs are separated slightly more than normal, producing a frontal plane movement [6,7].

These variations widen the body’s support and reduce propulsive force [8]. Increased sagittal pelvic tilt [9] can provoke hyperextension of the knees, weakening the knee extensor and flexor muscles and the hip extensors and abductors, and shortening some muscle groups, such as the adductors and external rotators of the hip and the plantar flexors of the ankle [10]. Consequently, the sacrum acquires a more horizontal position and the intervertebral space decreases [11].

During pregnancy, weight gain overloads the knees, ankles and feet and flattens the medial longitudinal plantar arch in the foot, which increases pressure in the midfoot area [12,13]. There is a greater tendency to suffer leg cramps, which are exacerbated by the increased demands made of the plantar flexors of the ankle [10].

In addition, foot posture is modified, becoming more pronated. In this respect, a recent study reported a change of 3.78 points in the Foot Posture Index [12,14]. The laxity of the plantar calcaneal-navicular ligament, together with the shortening of the posterior tibial tendon, can produce a flattening of up to 1 cm in the talus head, generating midfoot pronation [6]. This, together with the reduced height of the longitudinal arch, can produce a flattening in the entire foot architecture [13]. Furthermore, the physiological changes that take place in late pregnancy can lead to increased plantar pressure in the midfoot [15,16].

During the second and, especially, the third trimester of pregnancy, the eversion of the ankle decreases, which heightens the anterior inclination of the pelvis and the external rotation of the hip [17]. The centre of plantar pressure shifts to the rear and to the medial side. The mean static pressure of the second metatarsal joint (M2) and the third metatarsal joint (M3) increases, as does the contact area of the midfoot and the hindfoot. All phases of gait increase in duration and decrease in speed, altering stability and heightening the risk of falls. The kinematic and static characteristics of the lower limb return to the normal, non-pregnant state four months after the puerperium, according to Qichang Mei [18].

The aim of this study is to determine the morphological and postural changes that take place in the static foot during pregnancy and the puerperium. Prior studies have reported evidence of the physical changes that occur in this respect during pregnancy but not in the postpartum period. For this reason, we examine the changes recorded during pregnancy and consider whether they persist during the postpartum period.

## 2. Materials and Methods

This descriptive, observational, longitudinal study is based on a convenience sample of pregnant women, analysing data recorded at three time points: between weeks 9–13 of pregnancy, weeks 32–35 of pregnancy and weeks 4–6 after delivery.

The women included in the study were all in the first trimester of a single pregnancy (4–8 weeks), in the first pregnancy and aged under 40 years. Exclusion criteria were differences in length in the lower limbs; neurological disorder; severe trauma or surgery in the last six months affecting the lower extremities; or systemic disease affecting the locomotor system, toxemia, pre-eclampsia and multiple babies.

The study population was composed of 23 women, recruited at the Malvarrosa Primary Care Centre in Valencia (Spain). The following general characteristics were recorded: right-handed, average age 30.04 years (±4.4), height 1.63 m (±0.04), weight 69.1 kg (±18), BMI 25.9 kg/m^2^ in the first visit and foot size 38 (±1.2)

The study protocol complied with the established ethical principles for human research. Written informed consent for participation and publication was given by each patient, including for the publication of photographs. The study was approved by Universidad de Valencia (No. H1397032220515).

The dependent variables analysed were the Foot Posture Index, the Hernández-Corvo index (determined using a pedigraph) and anthropometric measure; the foot length was measured with a flexometer (Brico Tech, Elmira, 88 NY, USA) and delivery data. The data collection process was repeated at each review during the pregnancy and puerperium.

The Hernández-Corvo protocol (HCp) is a method for determining footprint characteristics. It is obtained by examining a pedigraph image of each foot, using the Tecniwork Pedrograph Plate (Innovaciones Tecniworks SL, Madrid, Spain), with the subject seated in a relaxed position. In the present study, the woman was instructed to place her heel against the pedograph plate edge without touching the plate surface. Once the foot was aligned over the pedigraph plate, the participant was asked to stand up firmly without wobbling and then to lift the opposite foot in order to fully load the tested foot. The HCp typifies the foot according to the footprint measurements obtained, on a six-point scale ranging from flat foot to severe cavus foot [19]

**Figure 1:**
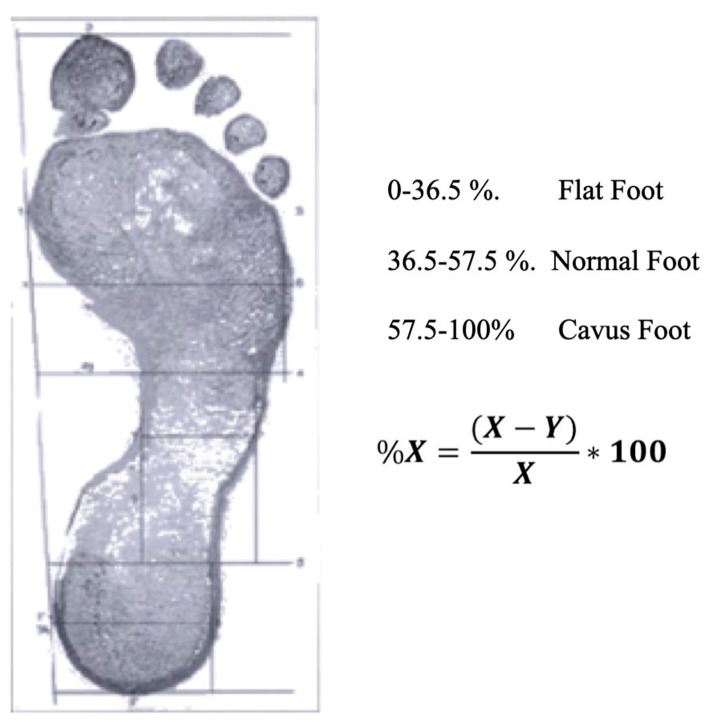
Classification of foot type according to the percentage of X calculation.

Three categories of foot type were established, with the following percentages of X: 0–36.5% flat, 36.5–57.5% normal and 57.5–100% cavus (Figure 1).

In every case, the Foot Posture Index (FPI) was assessed, with the participant barefoot, relaxed and standing on a 50 cm platform to facilitate visual and manual inspection. In addition to the three cardinal planes of motion, the FPI takes into account six items referencing the position of the forefoot, midfoot and hindfoot: (i) talar head palpation; (ii) symmetry of supra and infra-lateral malleolar curvature; (iii) inversion/eversion of the calcaneus; (iv) prominence of the talo-navicular joint; (v) height of the medial longitudinal arch; and (vi) ab/adduction of the forefoot [20].

The FPI was determined by a podiatrist with known high intra-rater reliability for FPI scoring (intraclass correlation coefficient [ICC] = 0.91–0.98), who was blinded to the purposes of the study and to the participant’s identity. This analysis was conducted with ten external participants, who were examined and then re-examined 24 h later. The FPI is a six-item clinical assessment tool used to evaluate foot posture. It has acceptable validity and good intra-rater reliability (ICC = 0.893–0.958) [21]. The FPI evaluates the multi-segmental nature of foot posture in all three planes and does not require the use of specialised equipment. Each item of the FPI is scored between −2 and +2, resulting in a total ranging from −12 (highly supinated) to +12 (highly pronated). The index items include talar head palpation, curves above and below the lateral malleoli, calcaneal angle, talonavicular bulge, medial longitudinal arch, and forefoot to hindfoot alignment. At each follow-up visit, the same protocol was applied. In all other respects, the protocol described by Redmond et al. was followed [22,23].

### 2.1. Procedure

At each of the time points established for this study (during weeks 9–13 and 32–35 of pregnancy and weeks 4–6 after delivery), the same researcher determined the FPI for each participant, the footprint according to the HCp, foot length and body weight.

Another researcher, who was blinded to the identity of each participant, entered this information into a database. The statistical analysis of these data was performed by a specialist who was not a member of the research group.

### 2.2. Statistical Analysis

The data were analysed using the R statistical software package. The pedigraph data are described using measures of dispersion, i.e., mean, standard deviation, minimum, maximum and variance. The normality of the distributions was assessed by the Shapiro–Wilk test since a non-normal distribution of data was consequently established. The data were paired (having been obtained from the same persons), and so statistical tests were applied to compare paired data. As the sample size was less than 30, non-parametric tests were used.

The measurements obtained at the three study times were compared by the Friedman test, contrasting the null hypothesis of equality of distribution or equality of medians against the alternative hypothesis that at least one of the medians is different and the effect size by Cohen’s d. If the differences between the three study times were significant according to the Friedman test, the measurement times were then compared two-by-two. In this respect, the comparison of two measurement times, two feet or two gait speeds was conducted using the Wilcoxon signed-rank test, which examines the null hypothesis of equality of distribution between two measurements, against the alternative hypothesis that the distribution is different between the two measurements. In every case, the level of statistical significance assumed was *p* = 0.05.

## 3. Results

Among the sample of 23 women, the mean age of these women was 30.45 (SD 5.1) years and the mean height was 1.63 m (SD 12.4). The average weight in the first trimester of pregnancy was 69.95 kg (SD 15.2), and the mean BMI was 24.4 kg/m^2^ (SD 3.9). In the third trimester, the corresponding values were 78.16 kg (SD 18.3) and 29.3 kg/m^2^ (SD 3.45), respectively, and in the puerperium, they were 73.7 kg (SD 8.7) and 27.4 kg/m^2^ (SD 2.18). Average body weight increased by nine kilograms during the third trimester. Twenty women had a higher BMI during the puerperium than in the first trimester. The differences in BMI (according to the Wilcoxon test) were significant between all periods: from the first to the third trimester *p* = 0.00009, from the third trimester to the puerperium *p* = 0.00009, and from the first trimester to the puerperium *p* = 0.003.

In more than two thirds of the women in the sample, foot posture changed during pregnancy and postpartum, first from supination to pronation and then, during the puerperium, from pronation to neutral. Hence, the change did not revert completely during the puerperium. Statistical analyses of foot posture, for each foot at the three measurement times, did not reveal significant differences between the feet (right foot, from neutral to pronator in period 2, *p* = 0.182, in period 3, *p* = 0.135; from neutral to supinator, in period 2, *p* = 0.741, in period 3, *p* = 1; from pronator to supinator, in period 2, *p* = 0.747, in period 3, *p* = 1; left foot, from neutral to pronator, in period 2, *p* = 0.221, in period 3, *p* = 0.125; from neutral to supinator, in period 2, *p* = 0.278, in period 3, *p* = 0.984; and from pronator to supinator in period 2, *p* = 0.184, in period 3, *p* = 1) (Table 1) effect size of 0.2 (small).

**Figure 2:**
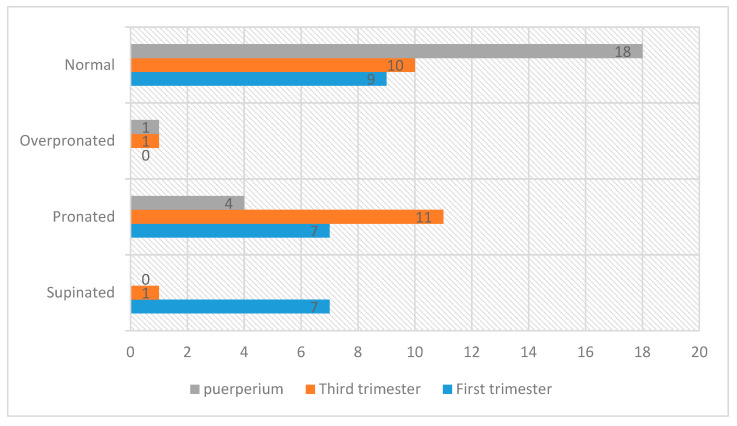
Changes in foot posture during pregnancy and puerperium (number of cases and type of foot posture).

During the third trimester, the pronator-type footprint appeared most frequently. It was predominantly neutral during the postpartum period (Figure 2).

**Figure 3:**
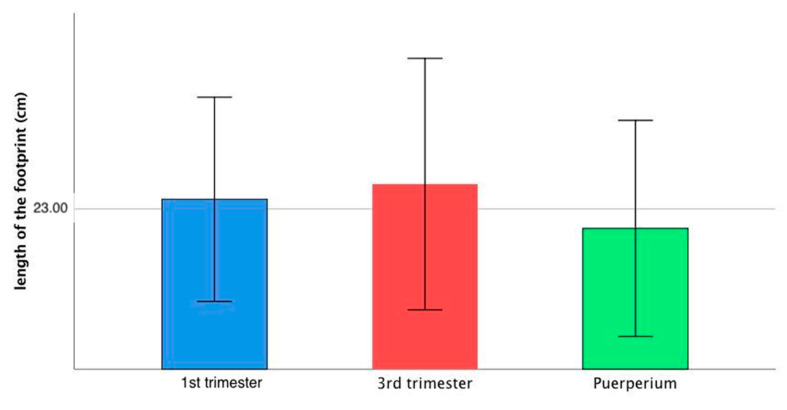
Changes in foot length during pregnancy and puerperium.

The length of the footprint remained basically unchanged, although there was a slight increase during the third trimester, which reverted after delivery. The difference, however, was not statistically significant (*p* = 0.084) (Figure 3).

**Figure 4:**
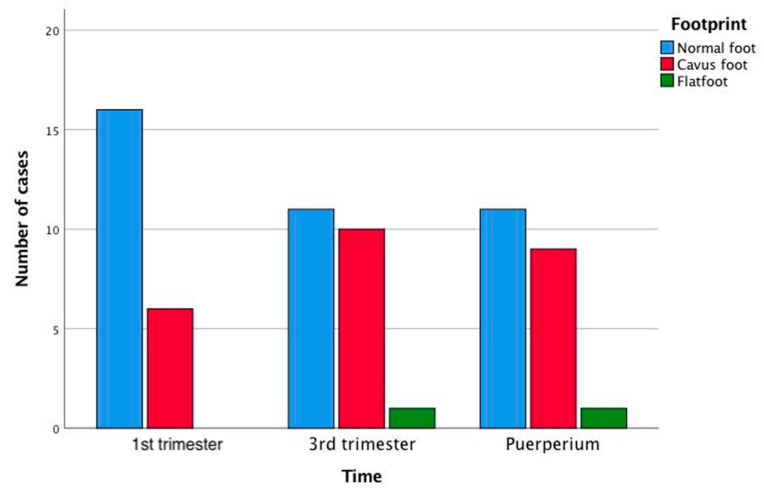
Changes in footprint during pregnancy and puerperium.

The pedigraph analysis to determine the percentage of X produced the following results: for the right foot: eleven normal feet, five normal/cavus, five cavus and one extreme cavus; for the left foot: six normal, five normal/cavus, six cavus, one strongly cavus, two extreme cavus and two flat. Statistically significant differences were obtained between the measurement times for the right foot, comparing the type of cavus and normal foot between the first and third trimesters and between the first trimester and the puerperium (in both cases, *p* < 0.0001) (Figure 4). Analysis of the percentage of metatarsal width and of the measure complementary to the external arch shows that the plantar arch flattens during pregnancy.

## 4. Discussion

The aim of this study is to examine the morphological and postural changes in the static foot during pregnancy and the puerperium. The results obtained show that the length of the footprint does not vary significantly during pregnancy, although there is a slight increase during the third trimester, which normalises after delivery (with a tendency to statistical significance in the case of the right foot). The latter pattern is associated with morphological changes typical of pregnancy; however, it does not seem to correlate statistically with weight gain during pregnancy, nor with the weight of the infant at birth [12,14,24,25]. Nevertheless, increased foot length during pregnancy has been widely observed in studies of this question [12,24,25,26,27].

Analysis of the FPI values obtained reveals changes in foot posture during the time periods considered. Over two thirds of the women presented a change in the type of footprint during their pregnancy, and in general these changes did not reverse completely in the puerperium. The majority of the women in the sample had a pronator-type footprint during the third trimester of pregnancy and a predominantly neutral one during the postpartum period.

The FPI indicates very little variability between the right and left foot, but great variability between the measurement periods. In ten cases, the footprint changed between the first and third trimesters, and in nine it changed between the third trimester and the puerperium. During the puerperium, the changes reverted in only three cases. The highest degree of pronation was observed in the third trimester, which corroborates previous research [12], and in many cases the footprint remained highly pronated at the end of the pregnancy and during the puerperium. The neutral footprint was most frequently observed after delivery. An uninterrupted decrease in supination was observed between the third trimester and the postpartum period, indicative of a trend towards pronation during pregnancy and a return to a more neutral posture afterwards. Some authors have observed an association between the tendency to pronation in pregnancy and a decreased height of the arch of the foot [25]. This flattening was also observed in our study in the overall pattern of footprint measurements obtained. In prior research, a greater tendency to pronation has been observed in obese populations of children, adults and the elderly [28,29,30], which suggests that weight gain in itself, regardless of whether it is due to pregnancy, could be the cause of a greater tendency to pronation.

Changes in the height of the arch of the foot (classed as normal, flat, cavus and intermediate categories according to the values of the percentage of X, taken as a discrete variable) were observed throughout the pregnancy, in both feet. However, statistically significant differences were only observed for an increase in the percentage of cavus feet in the third trimester and in the puerperium for the right foot, compared to the first trimester (*p* < 0.0001), assuming three categories (cavus, normal and flat). These data coincide with the results for the percentage of X measured as a continuous variable. Thus, lower values of %X were observed in the third trimester than in the other two periods, with a tendency to significance for the left foot (*p* = 0.097). The Ay measurement, which is complementary to the external arch Y, reflects the degree of flatness of the foot. This value presents a tendency to statistical significance for the left foot (*p* = 0.07). This analysis suggests that in the third trimester, the plantar arch height is lower than in the first trimester and in the puerperium. This is in line with published findings according to which the height of the arch of the foot tends to decrease in both feet [12,25,31]. In this respect, some studies have even observed a degree of permanence in these changes after delivery [25].

### Study Limitations and Future Lines of Research

The main limitation of this study is that of the very limited sample size considered. Furthermore, the data were largely obtained during the spring and summer months in a region where temperatures are high during these seasons, and this circumstance may have affected the results obtained. The measure of the FPI we must keep in mind is that the edema can be a limitation of the outcome measure, especially in the third trimester [5]. Finally, it should be noted that no parallel analysis was performed on a control population of non-pregnant women in order to neutralise possible measurement errors and to exclude the possibility that the variability observed may be implicit in the population.

As interesting areas for future research, we intend to study pedigraphs not only in the static foot but also in a dynamic state, and to include measurements of the plantar arch.

Our findings regarding changes in foot posture and characteristics during pregnancy and the puerperium enhance our understanding of these questions and may be applied in clinical practice, footwear recommendations and podiatric follow-up to prevent possible injuries.

## 5. Conclusions

Foot length increases during the third trimester of pregnancy and normalises during the puerperium. Changes in foot posture are apparent throughout the pregnancy. During the third trimester, pronation is predominant, and the posture returns to neutrality in the postpartum period. During pregnancy, the plantar arch flattens, but this condition changes during the puerperium. In consequence, there is a significant increase in the presence of cavus feet in the third trimester and in the puerperium.

## Figures and Tables

**Table 1:** Foot characteristics according to Foot Posture Index (FPI) and Hernández-Corvo protocol (HCp) index.

	First Trimester		Third Trimester		Puerperium		*p*-Value
	Mean	SD	Mean	SD	Mean	SD	
FPI	2.76	4.33	5.81	3.18	3.85	2.83	*p* < 0.0001
HCp index	57.78	16.33	57.46	15.04	61.37	17.06	*p* < 0.0001

## Data Availability

The data that support the findings of this study are available from the corresponding author upon reasonable request.
